# Genetic variation in disease resistance in *Drosophila* spp. is mitigated in *Drosophila sechellia* by specialization to a toxic host

**DOI:** 10.1038/s41598-023-34976-1

**Published:** 2023-05-13

**Authors:** Liam O’Malley, Jonathan Wang, Matthew Nikzad, Huiyu Sheng, Raymond St. Leger

**Affiliations:** Department of Entomology, University of Maryland, College Park, MD 20742 USA

**Keywords:** Evolution, Microbiology

## Abstract

We found that *Drosophila* species vary in their susceptibility to the broad-spectrum entomopathogen, *Metarhizium anisopliae* (strain Ma549). Generalist species were generally more resistant than dietary specialists, with the cactophilic *Drosophila buzzatii* and *Drosophila sechellia,* a specialist of the *Morinda citrifolia* (Morinda) fruit, being most susceptible. Morinda fruit is reported to be toxic to most herbivores because it contains Octanoic Acid (OA). We confirmed that OA is toxic to *Drosophila* spp., other than *D. sechellia,* and we also found that OA is highly toxic to entomopathogenic fungi including Ma549 and *Beauveria bassiana*. *Drosophila* *sechellia* fed a diet containing OA, even at levels much less than found in Morinda fruit, had greatly reduced susceptibility to Ma549. This suggests that specializing to Morinda may have provided an enemy-free space, reducing adaptive prioritization on a strong immune response. Our results demonstrate that *M. anisopliae* and *Drosophila* species with divergent lifestyles provide a versatile model system for understanding the mechanisms of host–pathogen interactions at different scales and in environmental context.

## Introduction

The sub genus *Drosophila* is the most intensively studied species assemblage in insects and includes some of the best-studied model organisms in ecological and evolutionary research. It has traditionally been subdivided into clades or groups including the *virilis*-*repleta* radiation (or the *repleta* clade) and the *melanogaster* radiation^1^. The original *repleta* clade radiation likely occurred in South America coincident with the evolution of Opuntia cacti^2^. Many current members of the *repleta* clade are generalists, including the necrotic fruit and animal feces feeder *D. repleta*. However, most *repleta* group species have larvae that feed on decaying cacti in arid or semiarid regions. Opuntia use is common throughout the phylogeny (e.g., *D. buzzatti* and the basal species *D. navojoa*), but several lineages, including *D. mojavensis,* have independently shifted in host use to the more chemically complex columnar cacti^3^. *D. arizonae* split from *D. mojavensis* only 1.5 MYA, but shows much less host plant specialization, utilizing decaying fruits and vegetables as well as various cacti^4^.

With the exception of the cosmopolitan human commensals *D. melanogaster* and *D. simulans*, the members of the *melanogaster* subgroup are found only in Africa, and some are nutritional specialists including *D. sechellia* on toxic *Morinda citrifolia* fruit^5^ and the tropical rain forest *D. erecta* on screw-pine fruit^6^. *Drosophila* *simulans* and *D. melanogaster* are generalist necrotic food feeders in their global range, although in its ancestral South African range, *D. melanogaster* is a specialist for marula fruit^7^. The diverse lifestyles of *Drosophila* spp provide an evolutionary framework and valuable opportunities for examining questions related to adaptive host specialization. To date, most attention has focused on *D. sechellia*, as it specialized recently (< 3MYA) from a generalist ancestor, and is very closely related to the key model organism *D. melanogaster* as well as *D. simulans*^8^. Morinda fruit are toxic due chiefly to high levels (~ 1.2%) of the medium chain fatty acid octanoic acid (OA) which *D. sechellia* has evolved resistance to and preference for^9^. OA is toxic to many insects, including parasitoid wasps, and specialization on Morinda has provided *D. sechellia* larvae with a parasitoid free space, potentially explaining its lack of natural resistance to parasitoids^10^. These results with parasitoids indicate that the specialization of *D. sechellia* on Morinda has altered its ecological interactions.

*Metarhizium* fungi are ubiquitous pathogens of insects that are of interest as models for understanding pathogen-host interactions and as biocontrol agents for insect pests^11,12^. Many *Metarhizium* species are capable of infecting and killing a wide range of insects, yet how they interact with the different genetic backgrounds and behaviors of their hosts is poorly understood^12^. Here, we deploy the broad-spectrum pathogen, *M. anisopliae* (strain Ma549) and nine species of *Drosophila* fruit flies to initiate studies on host pathogen interactions at ecological, and spatiotemporal scales. We show that plant host specialists are usually more susceptible than generalists in laboratory assays. However, we also report that OA is highly toxic to entomopathogenic fungi and in its presence *D. sechellia* is protected from Ma549. This extends the impact of OA from parasitoids to pathogens, and confirms the importance of considering host pathogen interactions in the environment in which they interact.

## Materials and methods

### Organisms and growth

*Metarhizium anisopliae* ARSEF 549 (Ma549), *Metarhizium robertsii* ARSEF 2575 and *M. robertsii* ARSEF 14447 were obtained from the U.S. Department of Agriculture Entomopathogenic Fungus Collection in Ithaca, N.Y. *Beauveria bassiana* 80.2 (Bb80.2) was kindly donated by George Dimopoulos (Johns Hopkins Bloomberg School of Public Health). For most experiments we used a line of Ma549 transformed to express GFP^12^ Cultures were maintained on potato dextrose agar (PDA) at 20 °C. Fungal cultures were moved from − 80 °C stock tubes 10–14 days before each bioassay and grown on potato dextrose agar (PDA) at 27 °C.

Flies were obtained from the National *Drosophila* Species Stock Center (NDSCC) at Cornell University. Flies were chosen to represent a range of different dietary specificities, localities, and clades. From the *repleta* clade: *D. repleta* (stock number: 15084–1611.06, 15084–1611.09, 15084–1611.11, 15084–1611.13; diet: rotting fruit, vegetables, feces)*, D. navojoa* (15081–1374.11; rotting opuntia)*, D. mojavensis* (15081–1352.03, 15081–1271.35, 15081–1352.48; columnar or barrel cacti) and *D. arizonae* (15081–1271.28 (Arizona), 15081–1271.33 (Sonora), 15081–1271.36 (San Diego), 15081–1271.38, 15081–1271.39 (Mexico); diverse cacti, vegetables and fruits)*.* From the *melanogaster* clade we chose: *D. sechellia* (14021–0248.07, 14021–0248.21, 14021–0248.25, 14021–0248.27, 14021–0248.28, 14021–0248.31; specialist of *Morinda citrifolia*)*, D. erecta* (14021–0224.00; specialist of screw pine (*Pandanus* spp.) fruit*, D. buzzatti* (15081–1291.06; opuntia), *D. yakuba* (14021–0261.00, 14021–0261.40, 14021–0261.48, 14021–0261.49; rotting fruit generalists)*, D. simulans* (14021–0251.005, 14021–0251.190, 14021–0251.254, 14021–0251.261, 14021–0251.286, 14021–0251.302; rotting fruit generalists).

Cactophillic *Drosophila* were raised and bioassayed on a diet supplemented with ground up opuntia and bananas as described at NDSCC. Other fly lines were reared on cornmeal-molasses-yeast-agar medium with tegosept and propionic acid (Genesee Scientific). We let food cool for 5–7 min before mixing in 1.2% OA as otherwise the OA oozed out of the food drowning the flies. All flies were maintained at 20 °C.

### Toxicity measurements

To measure the toxicity of OA, flies 2–5 days old were distributed into tubes each containing ~ 30 flies (three tubes per fly line, 14 different fly lines). Tubes contained the standard media for each species with or without 1.2% OA. Flies were transferred to fresh medium every 24 h for up to 14 days, and mortality was counted every 12 h.

To measure the toxicity of OA to fungi, we used 0.1% yeast extract medium supplemented with varying concentrations of OA (controls contained no OA). Medium was distributed in 2  ml aliquots into 35 mm petri dishes and inoculated (final concentration 5 × 10^4^ spores ml^−1^) with one of the four fungal strains (*M. anisopliae* ARSEF 549, *M. robertsii* ARSEF 2575, *M. robertsii* ARSEF 14447 and *B. bassiana * 80.2). These plates were maintained at 20 °C and inspected 18- and 40-h post-inoculation with an inverted microscope. Images of different fields of view were taken for each fungal strain and condition to cover at least 250 spores. From these images we calculated percent germination (defined as a germ tube at least half the length of the spore).

### Topical infection of *Drosophila* spp.

Bioassays using Ma549 tagged with green fluorescent protein (Ma549-GFP) were performed on *Drosophila* spp. Twenty-four hours prior to infection, flies (5–10 days old) were separated by sex and allocated (~ 30 flies per line per sex per tube, 2–3 replicates) into either standard media (cornmeal-molasses-yeast-agar medium), standard media plus 0.3% OA or standard media plus 1.2% OA.

Flies were bioassayed using the method of^12^. Briefly, flies were infected by vortexing for ~ 10 s with a spore suspension (2.5 × 10^4^ spores/ml of water) produced from 10-day old Ma549-GFP plates. Control flies were treated with water alone as a control for the bioassay process, and as previously reported there were no differences in longevity between untreated and water treated flies^13^. Infected and control flies were maintained at 27 °C (85% humidity), and transferred to fresh media every 24 h for up to 14 days. The flies were maintained on whichever media they had been placed on 24 h prior to infection. At intervals post infection, four flies from each tube (i.e., segregated per line, sex and growth media), were isolated and knocked out using the FlyNap Anesthetic Kit. Pictures of flies were then taken under bright-field and fluorescence lighting conditions using a fluorescence microscope. Mortality was counted every 12 h, and mean survival times (MSTs) or LT_50_’s were analyzed using R, while the percent survival calculations were performed in google sheets.

## Results

### *Drosophila spp.* vary in their susceptibility to infection

**Figure 1:**
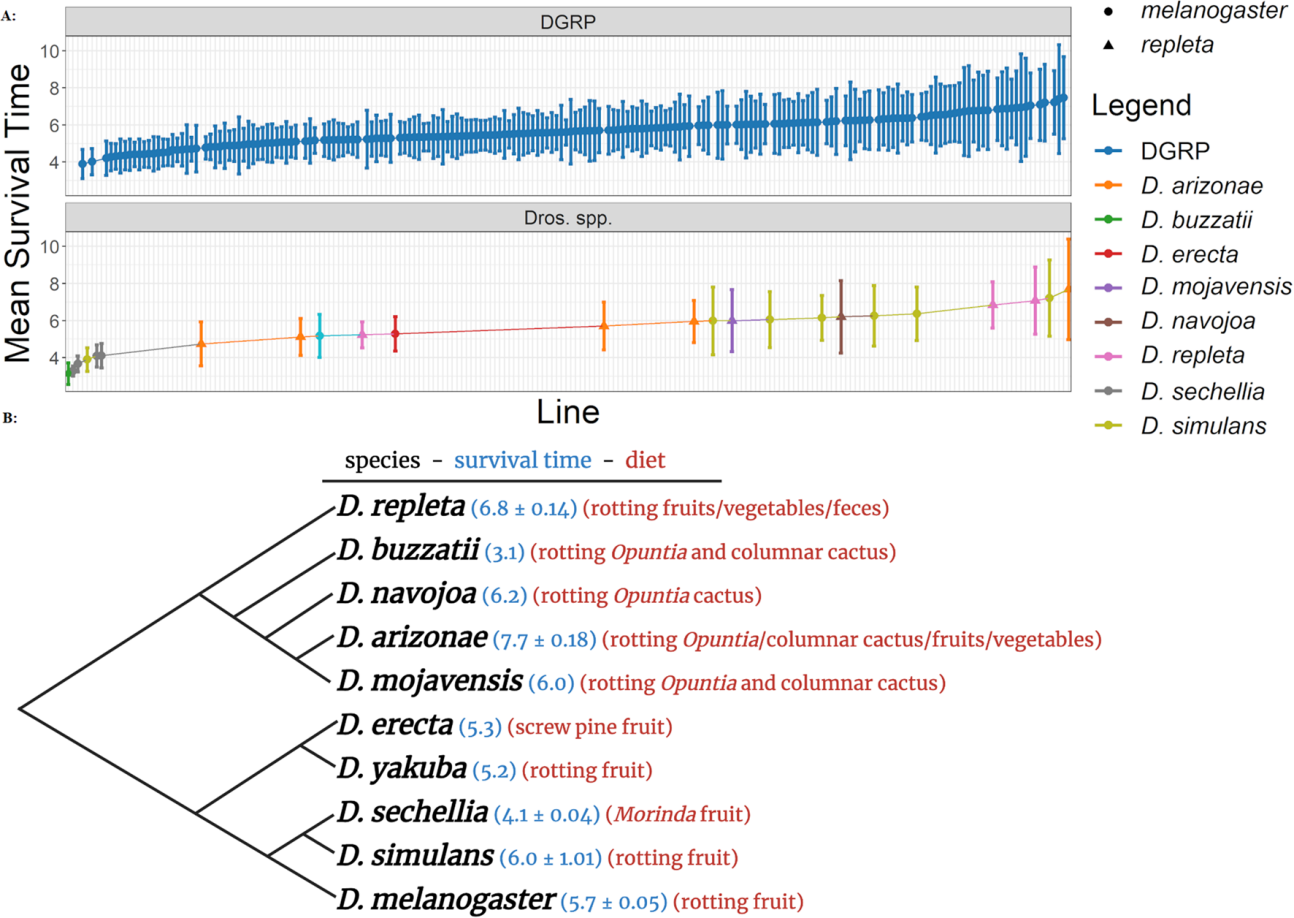
Survival of various *Drosophila* species after Ma549 infection. (**a**) Graph displaying mean survival times of 188 *D. melanogaster* lines in the Drosophila Genetic Reference Panel (top) as well as various species of *Drosophila* (bottom). (**b**) Phylogenetic relationship of the various *Drosophila* species alongside the mean survival time + /− standard deviation.

We examined the ability of Ma549, a broad host range insect pathogen, to infect and kill nine phylogenetically and ecologically diverse *Drosophila* species. We found that the mean survival time (MST) of the non-*melanogaster* fly species infected with Ma549 is 5.47 days with a range of 3.14–7.69 days. This is similar to the variation found in 188 lines from the DGRP (*D. melanogaster* Genetic Reference Panel) from Raleigh, North Carolina (ranges from 3.9 to 7.54 days)^13^ (Fig. 1a,b, Supplementary Table S1). The five most resistant lines were *D. arizonae* (15081–1271.28, MST = 7.691), *D. simulans* (14021–0251.286, 7.219), *D. repleta* (15084–1611.06, 7.08), *D. repleta* (15084–1611.09, 6.842), and *D. simulans* (14021–0251.261, 6.37), but overall survival times did not differ significantly (p = 0.2573, Welch’s two-tailed t-test t = 1.16) between the *D. melanogaster* (µ = 5.197 ± 1.257, n = 13, Shapiro–Wilk p = 0.31) and *repleta* clades (µ = 5.794 ± 1.245, n = 11, Shapiro–Wilk *p* = 0.39). Specialists (µ = 4.255 ± 1.115, n = 7, Shapiro–Wilk *p* = 0.29) with narrow diets were more susceptible (p = 0.006 Welch’s two-tailed t-test t = 3.57) than generalists (µ = 5.97 ± 0.953, n = 17, Shapiro–Wilk *p* = 0.94). Lines of *D. mojavensis* are hard to classify by dietary specificity as though cactophillic they feed on many types of cactus. However, including these lines as specialists, generalists or omitting them altogether did not change the significant t-test results for generalists versus specialists. A single line of *D. buzzatii* was the most susceptible to Ma549 (MST 3.1 ± 0.04) with four lines of *D. sechellia* all being highly susceptible showing an average MST of 3.79 ± 0.198.

### Octanoic acid toxicity across various fly species

It was previously reported that the Morinda fruit component Octanoic Acid (OA) lacks toxicity to *D. sechellia*, despite being toxic to its sister species, *D. melanogaster*^14^. To confirm this, and further explore differential toxicity levels, we monitored the survival of multiple lines of seven *Drosophila* species placed on food containing 1.2% OA. To represent the range of susceptibility to Ma549 previously found in 188 *D. melanogaster* DGRP lines (Fig. 1a,b, Supplementary Table S1), *D. melanogaster* was represented by the third most susceptible line DGRP_321 (LT_50_ ± SD = 4.21 ± 0.923 days, n = 115), and the most resistant line DGRP_808 (LT_50_ ± SD = 7.471 ± 2.2161 days, n = 155)^13^.

**Figure 2:**
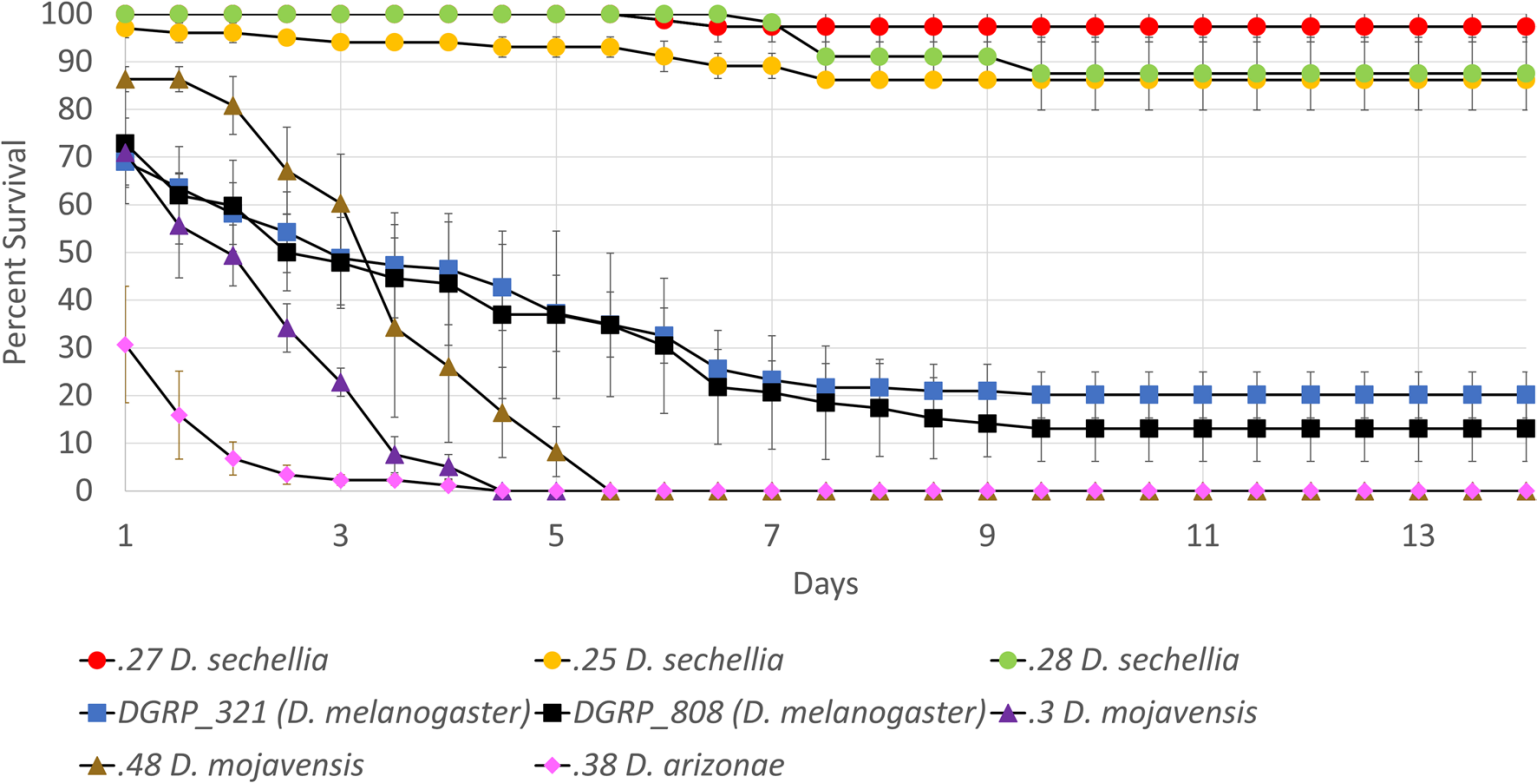
The percent survival of each *Drosophila* line on 1.2% OA food over 14 days. Note that the lines where there was 100% mortality by day 2 are not shown (.48 *yakuba*, .49 *yakuba*, .254 *simulans*, .13 *repleta*, .06 *repleta*, and .33 *arizonae*). Three lines shown on the graph, .3 *mojavensis*, .48 *mojavensis*, and .38 *arizonae*, overlap at 0% survival. Each data point represents the results from three replicates plus standard errors. The replicates ranged from 17 to 49 flies.

Our results extended previous findings by confirming OA was toxic to species other than *D. sechellia*. In nine of the 11 non-*D. sechellia* lines all flies were dead within 2 weeks, with most flies knocked down within a day (Fig. 2). The two comparatively resistant lines were *D. melanogaster* with over 75% mortality over 2 weeks, as compared to less than 10% mortality in flies not exposed to OA. Less than 14% of flies in three *D. sechellia* lines were dead within 2 weeks, a similar level of mortality to flies not exposed to OA.

### Fungal infection and survival assay

**Figure 3:**
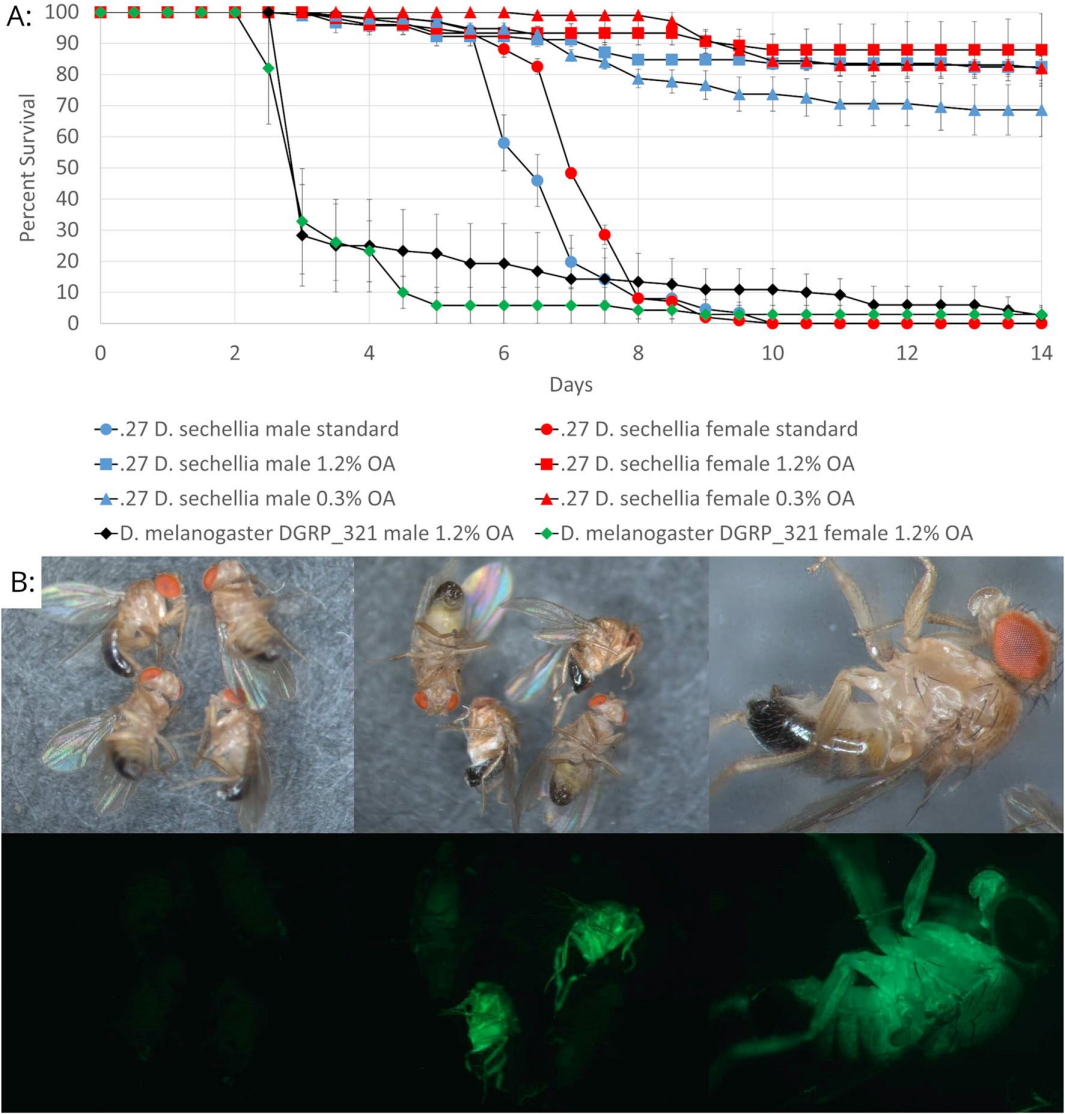
Results of Ma549 fungal infection on *D. sechellia* and *D. melanogaster* fly lines. (**a**) *D. sechellia* and *D. melanogaster* fly survival post infection on standard food and food plus 0.3% or 1.2% OA. For each strain 2–3 biological replicates were used with each *D. sechellia* replicate containing 26–54 flies. There was variation in the number of flies per *D. melanogaster* replicate as many died soon after being placed on OA food (before the onset of mycosis) and these were not included in the quantitative analysis. Error bars in the chart indicate standard errors of the averaged replicates. Additional data is available in Supplementary Tables S2–S5. (**b**) Growth of GFP-expressing Ma549 can be visualized in *D. sechellia* flies 4–6 days post-infection. Pictures are flies exposed to 1.2% OA food (left) or normal food (middle and right). The same flies were visualized with both bright-field (top) and epifluorescence, with filters set to detect GFP fluorescence (bottom).

*Drosophila sechellia* and *Drosophila melanogaster* were placed on media containing various concentrations of OA, and 24 h later were infected with Ma549 expressing GFP to facilitate detection of fungus within the host. The progress of infection with and without OA was followed for 2 weeks. In the absence of OA, *D. sechellia* died at a similar rate as the very susceptible *D. melanogaster* DGRP_321 line, and significantly faster (in males t = 8.4, *p* = 0.0035 and in females t = 4.7713, *p* = 0.0412) than the resistant *D. melanogaster* line DGRP_808. Supplementing food with 1.2% OA increased survival of *D. sechellia* from 0% (on standard food) to > 75% survival 14 days post-infection (Fig. 3a). Reducing the OA concentration to 0.3% still provided robust protection (> 65% survival) compared to standard medium (male: 6.49 ± 0.24, female: 6.98 ± 0.14) (Fig. 3a). Further data on these infections can be found in Supplementary Tables S2–S5.

The Ma549 transformant expressing GFP (Ma549-GFP) allowed us to track infections in *D. sechellia* with and without exposure to OA (Fig. 3b). Flies were considered infected if Ma549 was visible inside the body. In the absence of OA ~ 50% of flies showed extensive GFP-fluorescence within 5 days post-infection, coincident with mortality, and this rose to 100% after 8 days. No flies fluoresced in the presence of OA, indicating that OA blocked infection.

### Octanoic acid inhibition of fungal growth

**Figure 4:**
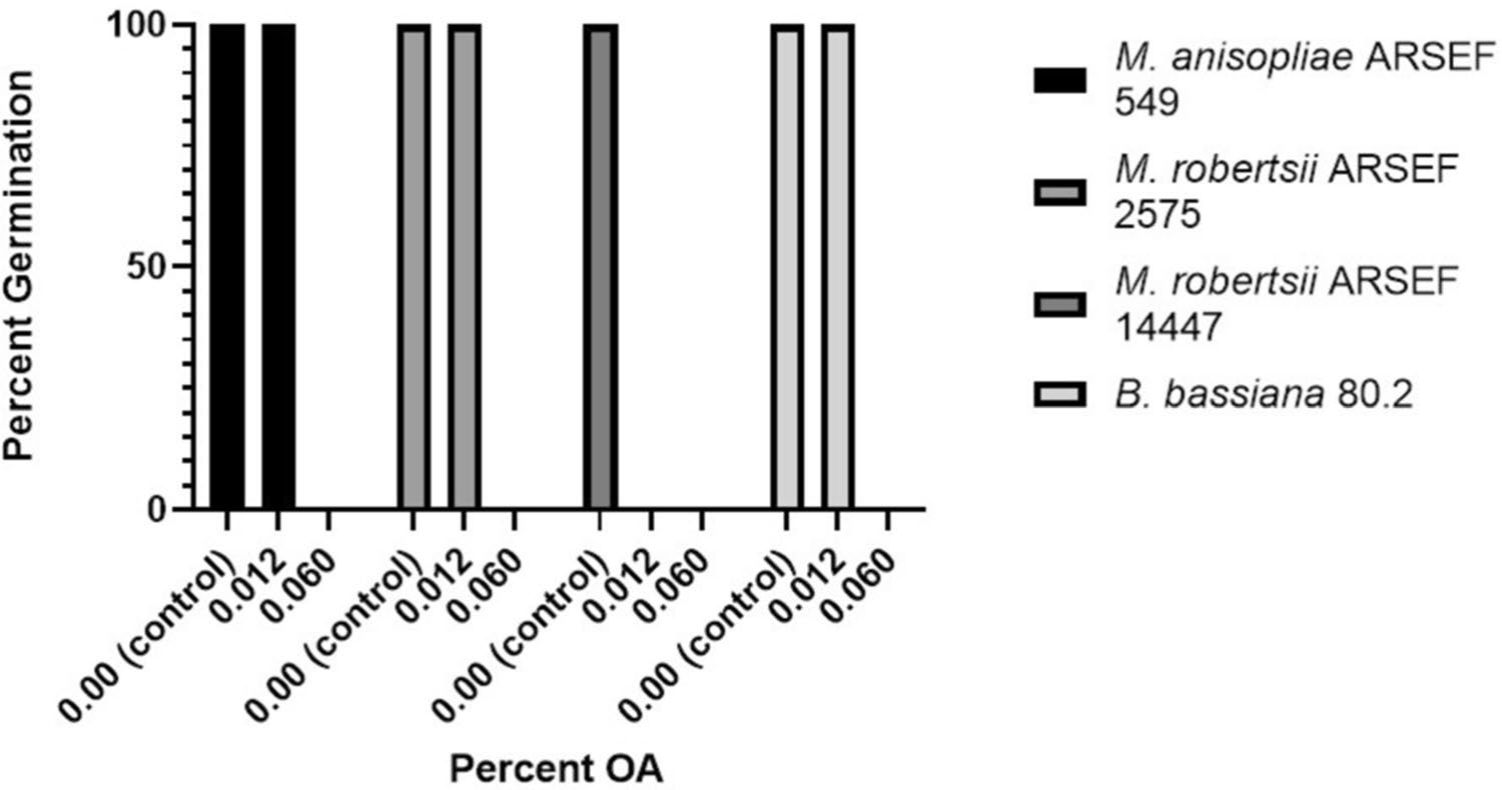
Effects of OA on Fungal Germination. Percent of fungal spores that germinated after 40 h on 0.1% Yeast Extract Medium with varying percentages of OA (0%, 0.012%, 0.06%) (Supplementary Table S6). All strains displayed complete inhibition of germination at 0.06% OA, a concentration 20 times lower than that found in the Morinda fruit.

The toxicity of OA could either directly inhibit the Ma549, for example preventing germination and growth, or indirectly effect the fungus through boosting *D. sechellia* host defenses. We tested four fungal strains (*M. anisopliae* ARSEF 549, *M. robertsii* ARSEF 2575, *M. robertsii* ARSEF 14447 and *B. bassiana* 80.2) in 0.1% yeast extract containing varying concentrations of OA. We determined that OA completely inhibited germination of all fungi tested at or above a concentration of 0.06% OA after 40 h (Fig. 4, further data Supplementary Table S6), i.e., at 20-fold lower levels than the concentration of OA in mature Morinda.

## Discussion

Fungi, such as *M. anisopliae* cause the majority of insect disease and play a crucial role in natural ecosystems^15^. *Metarhizium* *anisopliae* and *Beauveria bassiana* are also being developed as biocontrol agents against fruit fly pests^16^. As most *M*. *anisopliae* strains, including Ma549 used in this study, have a broad host range they are unlikely to be engaging in a strict coevolutionary arms race with a particular *Drosophila* population. Using *M*. *anisopliae* in infection experiments thus allows us to study how hosts respond to a generalist fungal pathogen, and to assess if variability among host populations is present, possibly due to divergent life histories^13^.

Our study involved screening 31 lines representing nine diverse *Drosophila* spp for susceptibility to Ma549. We found variation in mean survival times within the *repleta* and *melanogaster* clades ranging from 3.1–7.7 to 3.3–7.2 days, respectively. Previous *D. melanogaster* bioassays screening 188 North Carolina DGRP lines revealed a range from 3.9 to 7.5 days^13^. Similar variation may be found in other species as lines of the generalists *D. arizonae*, *D. repleta* and *D. simulans* varied from highly resistant to susceptible on the scale set by the DGRP lines. The most susceptible of the 31 lines was our only representative of *D. buzzatti*. This species clearly merits further screening, but that will require capturing additional wild lines as it is not well represented in culture collections.

As we cannot sample the entire population for the total variation in all species, our approach is to estimate a baseline resistance for each species so that we can generate and test hypotheses related to environmental context and disease resistance. We previously speculated^13^ that flies with a specialized diet may tend to stay on their preferred host, and therefore encounter fewer different pathogens. In contrast, generalist flies may have a relatively high migration rate and consequentially be exposed to more diverse pathogens, and thus have been selected to show greater overall resistance. More lines of multiple *Drosophila* spp. will need to be bioassayed with Ma549 to confirm the generality of associations between fly dietary specificity and disease susceptibility, but the current study confirms they are linked in *D. sechellia*, albeit the link involves adaptation to a toxic host plant.

Adaptation to novel host plants is a major driver of insect diversification due to many factors that include accessing new food sources and changing the competitive dynamic among species^17^^,^^18^^,^^19^^,^^20^^,^^21^. The resistance of *D. sechellia* to the octanoic acid in Morinda likely provides this species with a unique ecological niche, minimizing potential competition with coexisting *Drosophila* species^9^. *Drosophila* *sechellia* shares a recent common ancestor with *D. melanogaster,* presumably acquiring resistance to OA as it specialized to Morinda^22^. It is of interest therefore that *D. melanogaster* lines were the only ones besides *D. sechellia* with survivors 14 days post exposure to OA, implying that the common ancestor may have been preadapted to this shift by some natural resistance to OA. However, resistance is not shared with other members of the *D. melanogaster* clade, as lines of *D. yakuba* and *D. simulans* were rapidly killed.

Another factor that may contribute to speciation on novel hosts is that these may allow organisms to escape from their natural enemies into “enemy-free space” (EFS)^23^. Most studies on the potential explanatory power of EFS in structuring ecological niches have focused on host use by parasitoids^24,25^. However, EFS is likely to be a much more general phenomenon. Thus, our study utilizing a ubiquitous broad host range pathogen has potential parallels with generalist predators of insect herbivores that can also select for restricted plant host range if by so doing the herbivores are protected to some degree via allelochemicals^26^.

In this study we found OA to be directly toxic to important insect pathogens, suggesting that *D. sechellia*’s shift to being a specialist of Morinda provides broad protection against many natural enemies. Relaxation of selective pressure could result in degeneration of the immune defenses^27^. The transcriptional response of *D. sechellia* to parasitoid attack is very different from that of the closely related *D. simulans*, but interestingly many of the differentially expressed genes are involved in killing microbes and not parasitoids^28^. This is consistent with our study by suggesting that with the relaxation of selective pressure the general (multipurpose) defense mechanisms of *D. sechellia* have evolved an altered response that will likely affect susceptibility to multiple pathogens and parasitoids.

## Supplementary Information

Supplementary Tables.

## Data Availability

All data generated or analyzed during this study are included in this published article (and its Supplementary Information files).
